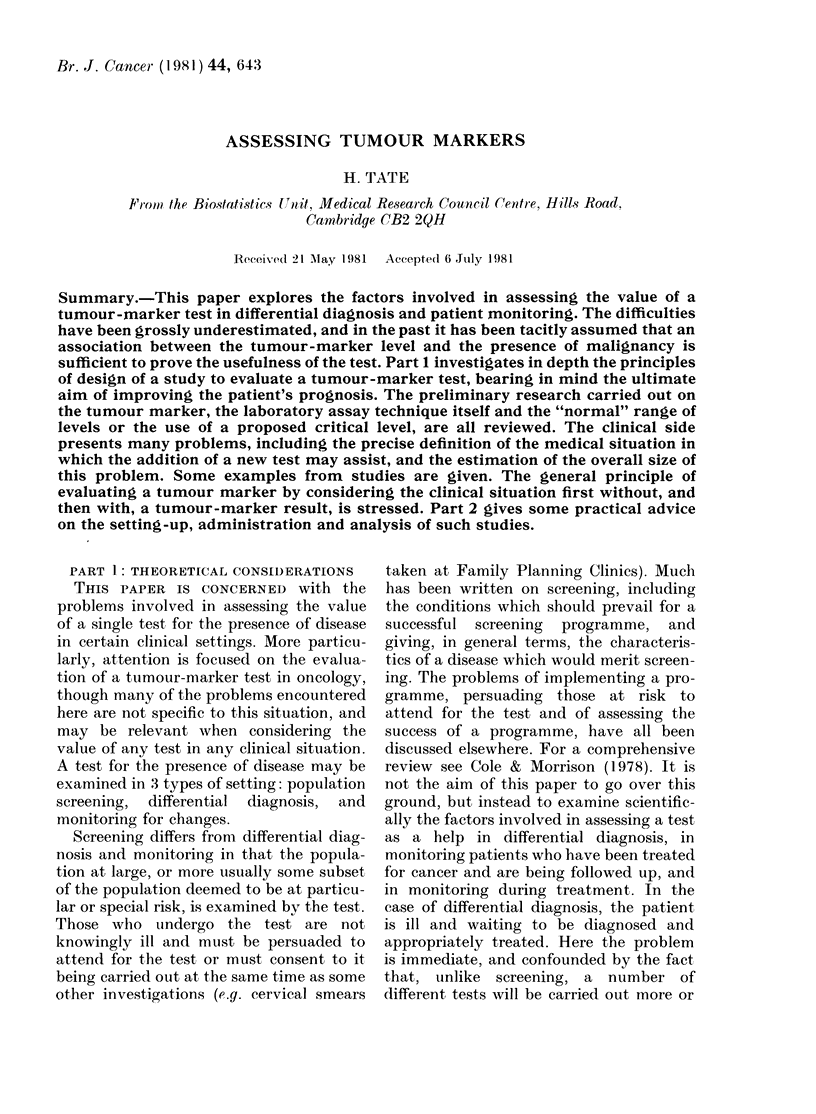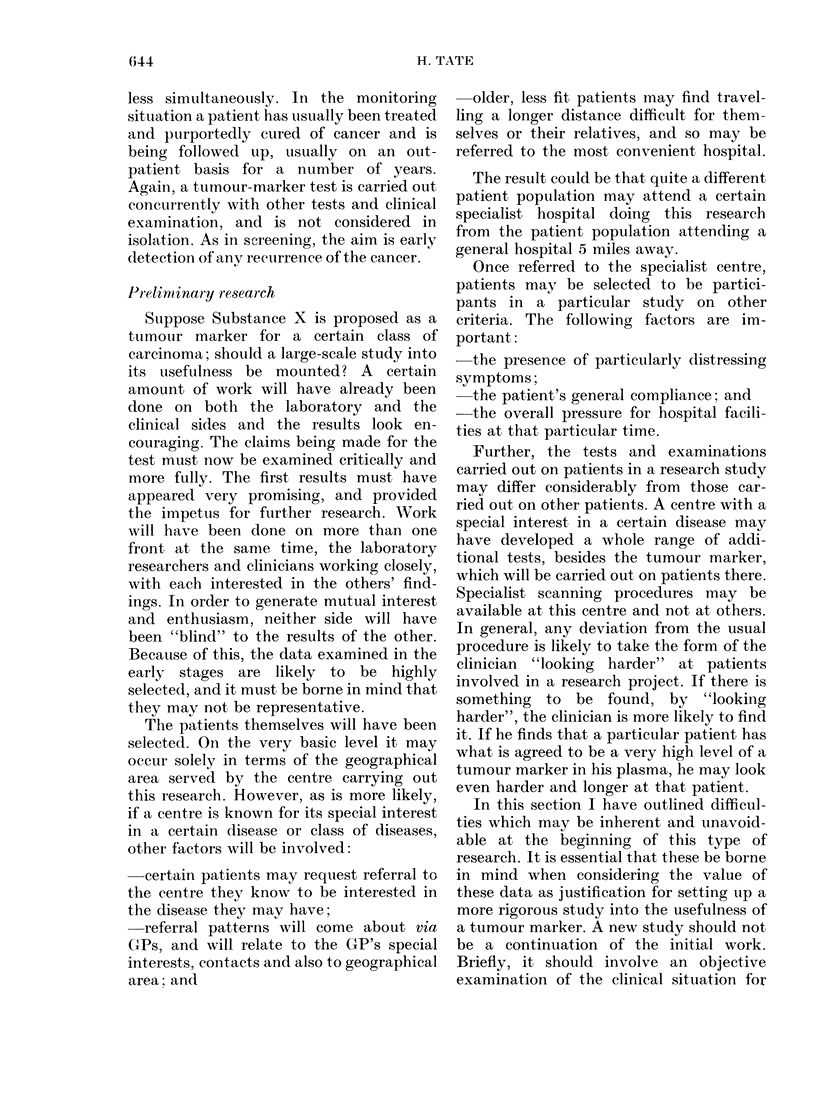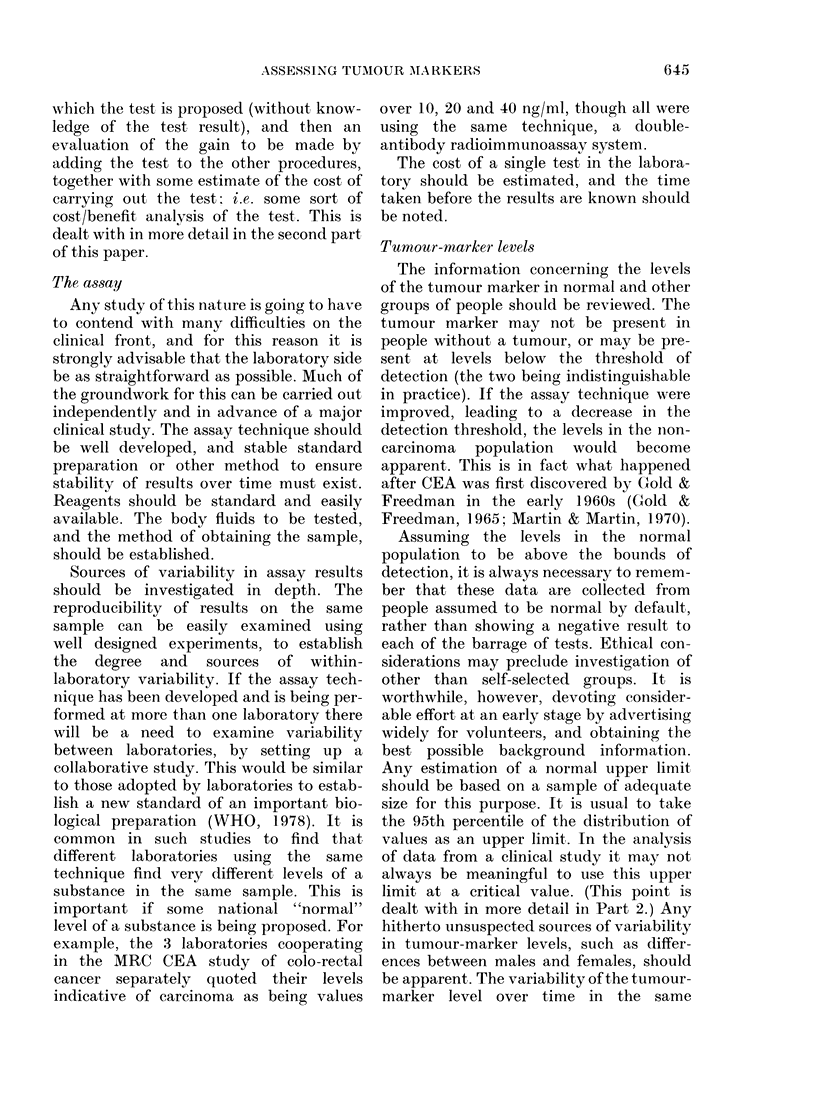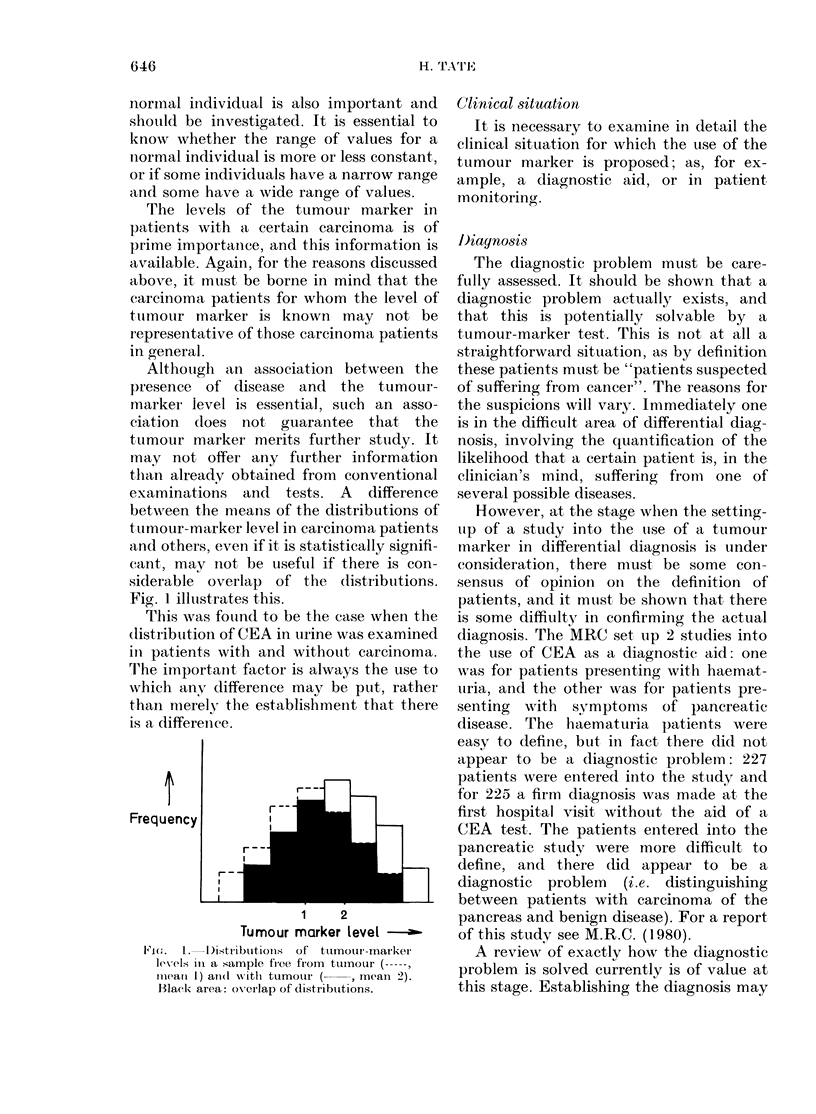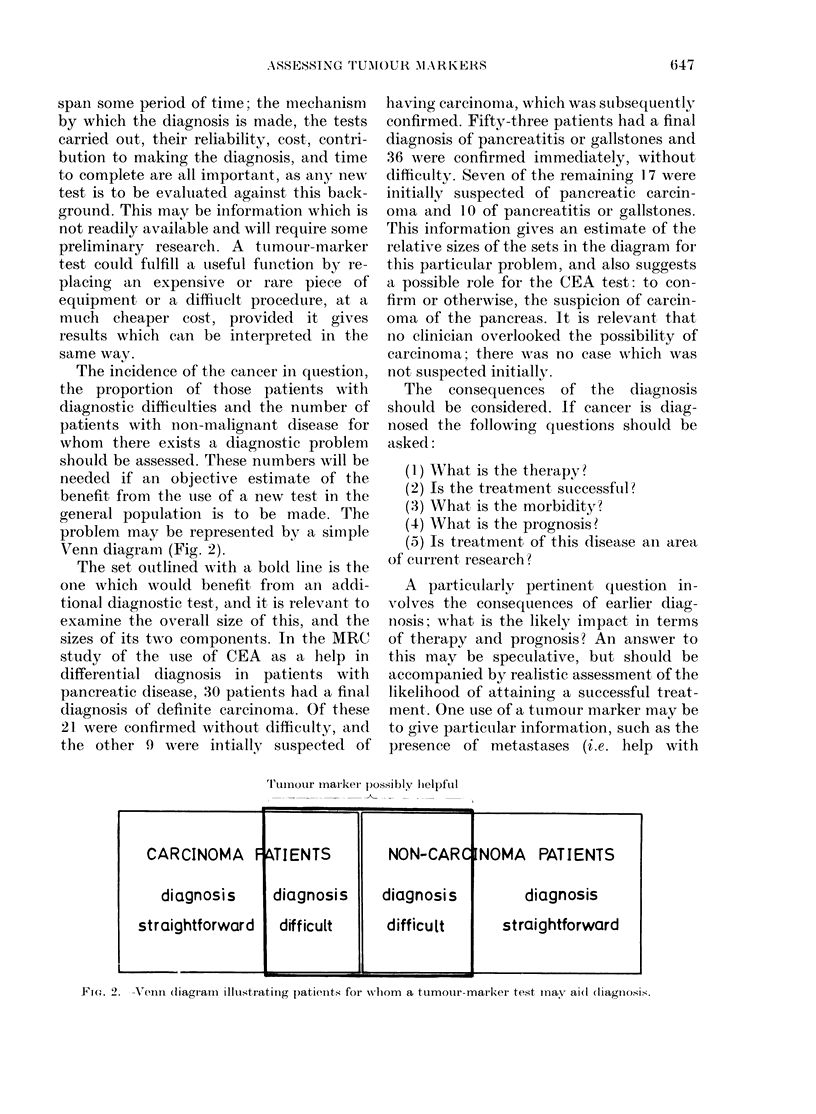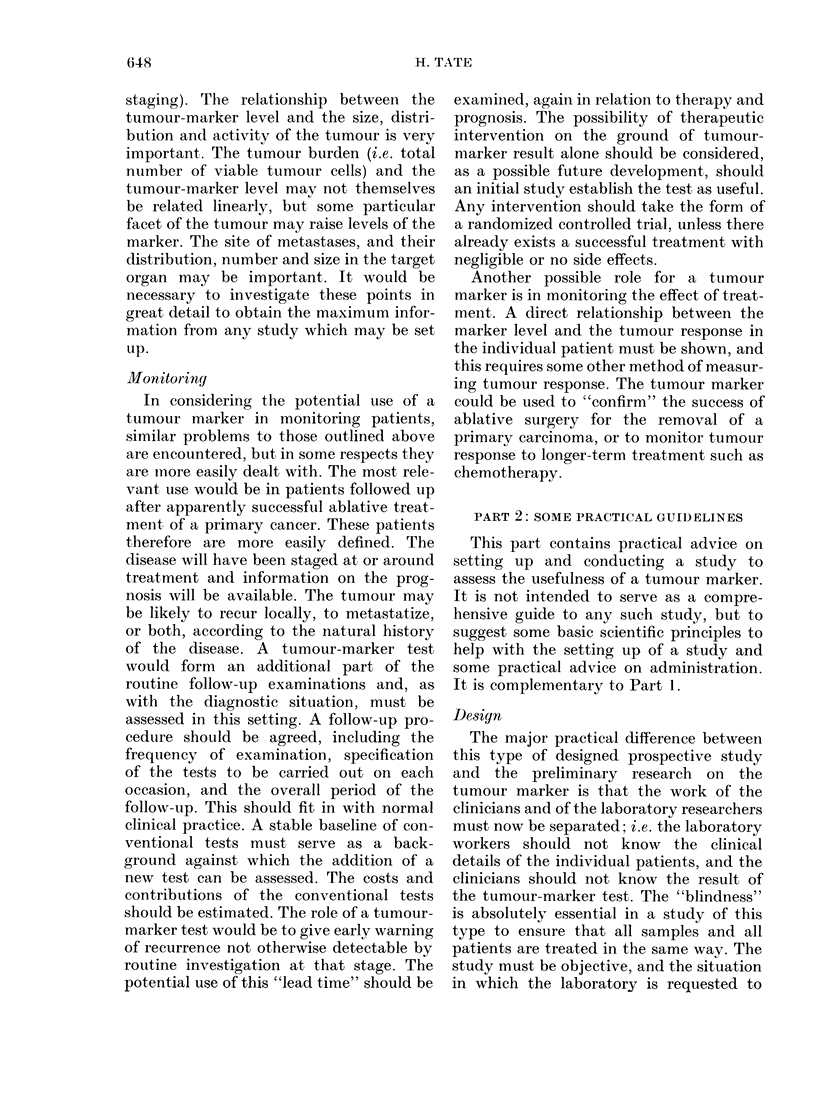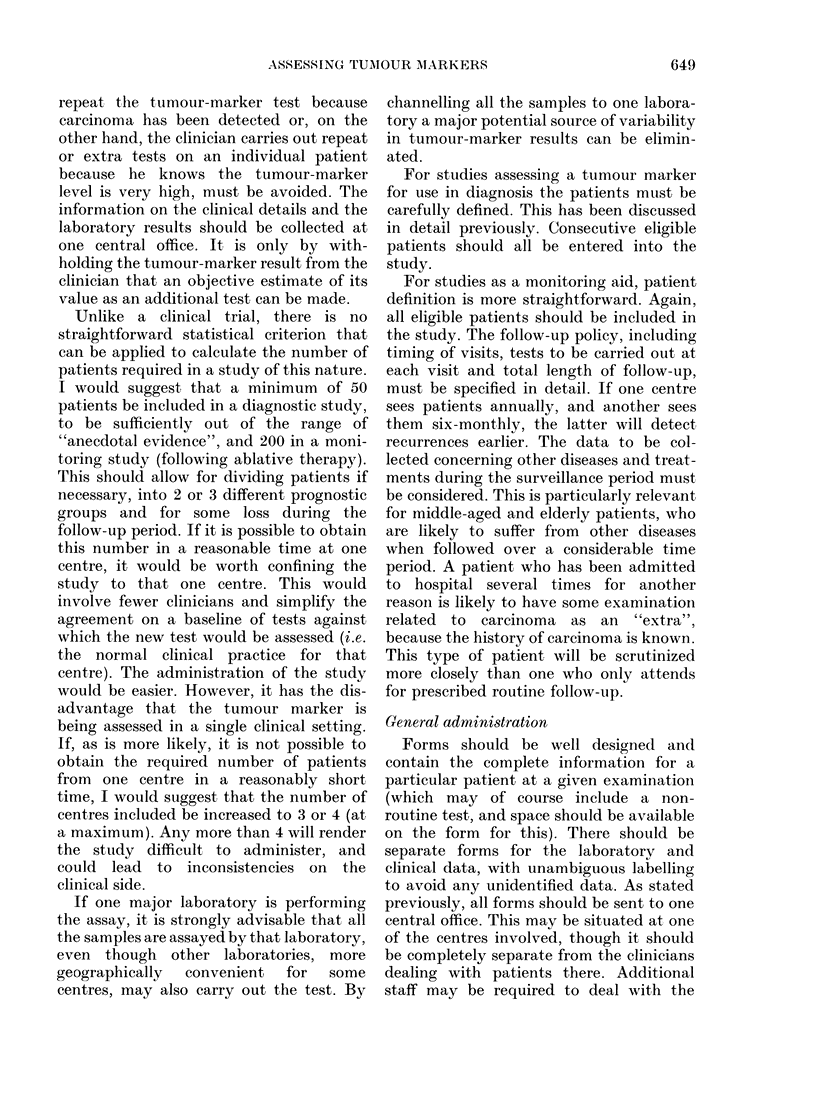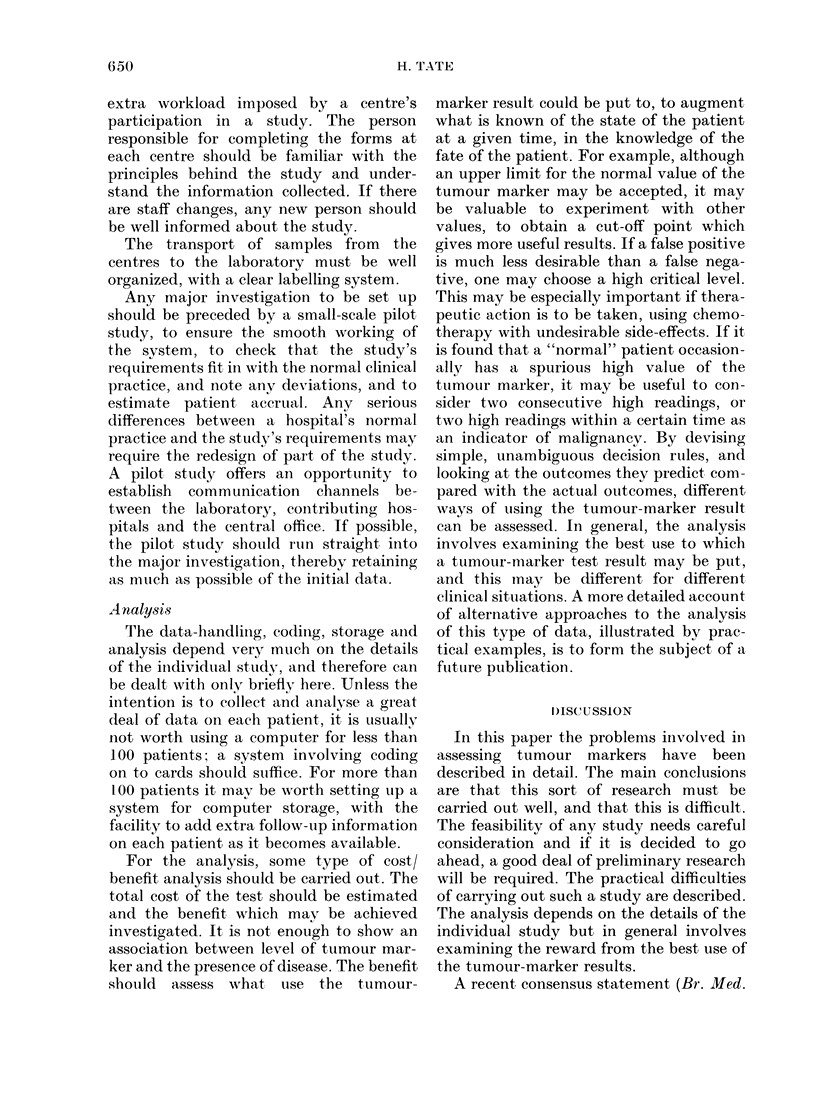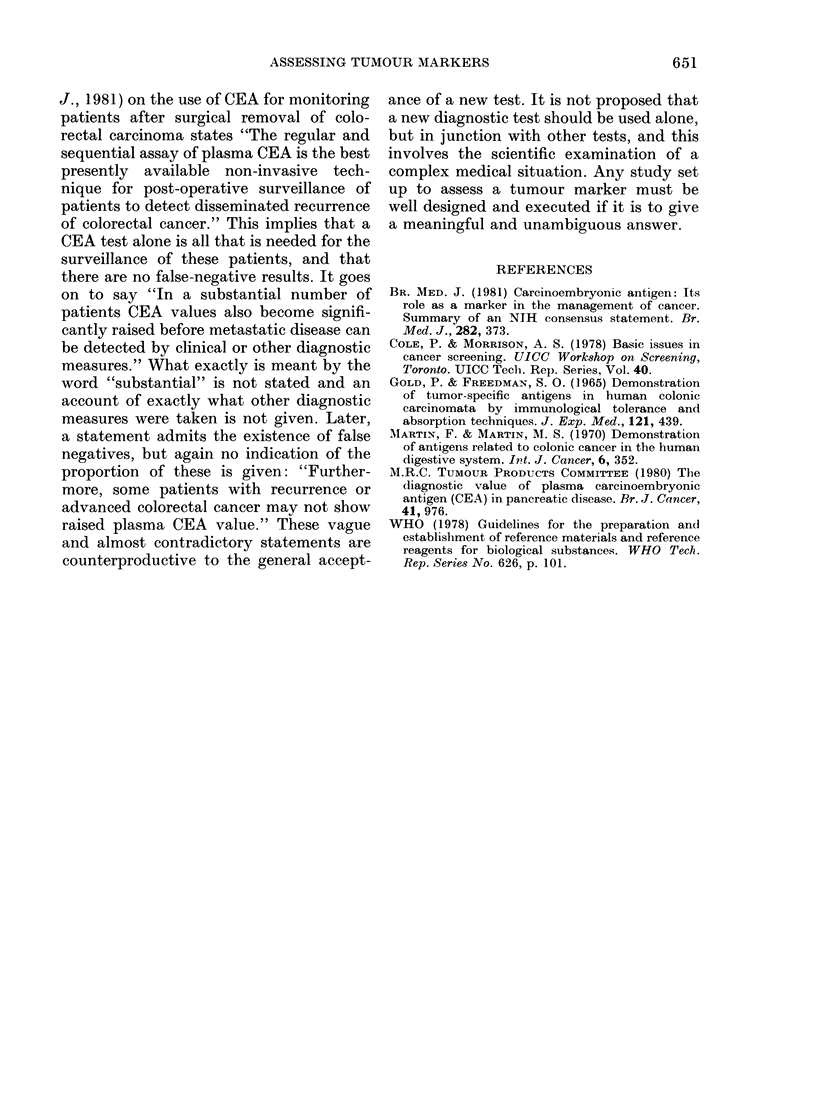# Assessing tumour markers.

**DOI:** 10.1038/bjc.1981.249

**Published:** 1981-11

**Authors:** H. Tate

## Abstract

This paper explores the factors involved in assessing the value of a tumour-marker test in differential diagnosis and patient monitoring. The difficulties have been grossly underestimated, and in the past it has been tacitly assumed that an association between the tumour-marker level and the presence of malignancy is sufficient to prove the usefulness of the test. Part 1 investigates in depth the principles of design of a study to evaluate a tumour-marker test, bearing in mind the ultimate aim of improving the patient's prognosis. The preliminary research carried out on the tumour marker, the laboratory assay technique itself and the "normal" range of levels or the use of a proposed critical level, are all reviewed. The clinical side presents many problems, including the precise definition of the medical situation in which the addition of a new test may assist, and the estimation of the overall size of this problem. Some examples from studies are given. The general principle of evaluating a tumour marker by considering the clinical situation first without, and then with, a tumour-marker result, is stressed. Part 2 gives some practical advice on the setting-up, administration and analysis of such studies.


*Br. J. Cancer* (1981) **44**, 643

### ASSESSING TUMOUR MARKERS

H. TATE

*From the Biostatistics Unit, Medical Research Council Centre, Hills Road, Cambridge CB2 2QH*

Received 21 May 1981 Accepted 6 July 1981

**Summary.—This paper explores the factors involved in assessing the value of a tumour-marker test in differential diagnosis and patient monitoring. The difficulties have been grossly underestimated, and in the past it has been tacitly assumed that an association between the tumour-marker level and the presence of malignancy is sufficient to prove the usefulness of the test. Part 1 investigates in depth the principles of design of a study to evaluate a tumour-marker test, bearing in mind the ultimate aim of improving the patient's prognosis. The preliminary research carried out on the tumour marker, the laboratory assay technique itself and the "normal" range of levels or the use of a proposed critical level, are all reviewed. The clinical side presents many problems, including the precise definition of the medical situation in which the addition of a new test may assist, and the estimation of the overall size of this problem. Some examples from studies are given. The general principle of evaluating a tumour marker by considering the clinical situation first without, and then with, a tumour-marker result, is stressed. Part 2 gives some practical advice on the setting-up, administration and analysis of such studies.**

### PART 1: THEORETICAL CONSIDERATIONS

THIS PAPER IS CONCERNED with the problems involved in assessing the value of a single test for the presence of disease in certain clinical settings. More particularly, attention is focused on the evaluation of a tumour-marker test in oncology, though many of the problems encountered here are not specific to this situation, and may be relevant when considering the value of any test in any clinical situation. A test for the presence of disease may be examined in 3 types of setting: population screening, differential diagnosis, and monitoring for changes.

Screening differs from differential diagnosis and monitoring in that the population at large, or more usually some subset of the population deemed to be at particular or special risk, is examined by the test. Those who undergo the test are not knowingly ill and must be persuaded to attend for the test or must consent to it being carried out at the same time as some other investigations (*e.g.* cervical smears taken at Family Planning Clinics). Much has been written on screening, including the conditions which should prevail for a successful screening programme, and giving, in general terms, the characteristics of a disease which would merit screening. The problems of implementing a programme, persuading those at risk to attend for the test and of assessing the success of a programme, have all been discussed elsewhere. For a comprehensive review see Cole & Morrison (1978). It is not the aim of this paper to go over this ground, but instead to examine scientifically the factors involved in assessing a test as a help in differential diagnosis, in monitoring patients who have been treated for cancer and are being followed up, and in monitoring during treatment. In the case of differential diagnosis, the patient is ill and waiting to be diagnosed and appropriately treated. Here the problem is immediate, and confounded by the fact that, unlike screening, a number of different tests will be carried out more or less simultaneously. In the monitoring situation a patient has usually been treated and purportedly cured of cancer and is being followed up, usually on an out-patient basis for a number of years. Again, a tumour-marker test is carried out concurrently with other tests and clinical examination, and is not considered in isolation. As in screening, the aim is early detection of any recurrence of the cancer.

*Preliminary research*

Suppose Substance X is proposed as a tumour marker for a certain class of carcinoma; should a large-scale study into its usefulness be mounted? A certain amount of work will have already been done on both the laboratory and the clinical sides and the results look encouraging. The claims being made for the test must now be examined critically and more fully. The first results must have appeared very promising, and provided the impetus for further research. Work will have been done on more than one front at the same time, the laboratory researchers and clinicians working closely, with each interested in the others' findings. In order to generate mutual interest and enthusiasm, neither side will have been "blind" to the results of the other. Because of this, the data examined in the early stages are likely to be highly selected, and it must be borne in mind that they may not be representative.

The patients themselves will have been selected. On the very basic level it may occur solely in terms of the geographical area served by the centre carrying out this research. However, as is more likely, if a centre is known for its special interest in a certain disease or class of diseases, other factors will be involved:

—certain patients may request referral to the centre they know to be interested in the disease they may have;
—referral patterns will come about *via* GPs, and will relate to the GP's special interests, contacts and also to geographical area; and
—older, less fit patients may find travelling a longer distance difficult for themselves or their relatives, and so may be referred to the most convenient hospital.

The result could be that quite a different patient population may attend a certain specialist hospital doing this research from the patient population attending a general hospital 5 miles away.

Once referred to the specialist centre, patients may be selected to be participants in a particular study on other criteria. The following factors are important:

—the presence of particularly distressing symptoms;
—the patient's general compliance; and
—the overall pressure for hospital facilities at that particular time.

Further, the tests and examinations carried out on patients in a research study may differ considerably from those carried out on other patients. A centre with a special interest in a certain disease may have developed a whole range of additional tests, besides the tumour marker, which will be carried out on patients there. Specialist scanning procedures may be available at this centre and not at others. In general, any deviation from the usual procedure is likely to take the form of the clinician "looking harder" at patients involved in a research project. If there is something to be found, by "looking harder", the clinician is more likely to find it. If he finds that a particular patient has what is agreed to be a very high level of a tumour marker in his plasma, he may look even harder and longer at that patient.

In this section I have outlined difficulties which may be inherent and unavoidable at the beginning of this type of research. It is essential that these be borne in mind when considering the value of these data as justification for setting up a more rigorous study into the usefulness of a tumour marker. A new study should not be a continuation of the initial work. Briefly, it should involve an objective examination of the clinical situation for which the test is proposed (without knowledge of the test result), and then an evaluation of the gain to be made by adding the test to the other procedures, together with some estimate of the cost of carrying out the test; *i.e.* some sort of cost/benefit analysis of the test. This is dealt with in more detail in the second part of this paper.

*The assay*

Any study of this nature is going to have to contend with many difficulties on the clinical front, and for this reason it is strongly advisable that the laboratory side be as straightforward as possible. Much of the groundwork for this can be carried out independently and in advance of a major clinical study. The assay technique should be well developed, and stable standard preparation or other method to ensure stability of results over time must exist. Reagents should be standard and easily available. The body fluids to be tested, and the method of obtaining the sample, should be established.

Sources of variability in assay results should be investigated in depth. The reproducibility of results on the same sample can be easily examined using well designed experiments, to establish the degree and sources of within-laboratory variability. If the assay technique has been developed and is being performed at more than one laboratory there will be a need to examine variability between laboratories, by setting up a collaborative study. This would be similar to those adopted by laboratories to establish a new standard of an important biological preparation (WHO, 1978). It is common in such studies to find that different laboratories using the same technique find very different levels of a substance in the same sample. This is important if some national "normal" level of a substance is being proposed. For example, the 3 laboratories cooperating in the MRC CEA study of colo-rectal cancer separately quoted their levels indicative of carcinoma as being values over 10, 20 and 40 ng/ml, though all were using the same technique, a double-antibody radioimmunoassay system.

The cost of a single test in the laboratory should be estimated, and the time taken before the results are known should be noted.

*Tumour-marker levels*

The information concerning the levels of the tumour marker in normal and other groups of people should be reviewed. The tumour marker may not be present in people without a tumour, or may be present at levels below the threshold of detection (the two being indistinguishable in practice). If the assay technique were improved, leading to a decrease in the detection threshold, the levels in the non-carcinoma population would become apparent. This is in fact what happened after CEA was first discovered by Gold & Freedman in the early 1960s (Gold & Freedman, 1965; Martin & Martin, 1970).

Assuming the levels in the normal population to be above the bounds of detection, it is always necessary to remember that these data are collected from people assumed to be normal by default, rather than showing a negative result to each of the barrage of tests. Ethical considerations may preclude investigation of other than self-selected groups. It is worthwhile, however, devoting considerable effort at an early stage by advertising widely for volunteers, and obtaining the best possible background information. Any estimation of a normal upper limit should be based on a sample of adequate size for this purpose. It is usual to take the 95th percentile of the distribution of values as an upper limit. In the analysis of data from a clinical study it may not always be meaningful to use this upper limit at a critical value. (This point is dealt with in more detail in Part 2.) Any hitherto unsuspected sources of variability in tumour-marker levels, such as differences between males and females, should be apparent. The variability of the tumour-marker level over time in the same normal individual is also important and should be investigated. It is essential to know whether the range of values for a normal individual is more or less constant, or if some individuals have a narrow range and some have a wide range of values.

The levels of the tumour marker in patients with a certain carcinoma is of prime importance, and this information is available. Again, for the reasons discussed above, it must be borne in mind that the carcinoma patients for whom the level of tumour marker is known may not be representative of those carcinoma patients in general.

Although an association between the presence of disease and the tumour-marker level is essential, such an association does not guarantee that the tumour marker merits further study. It may not offer any further information than already obtained from conventional examinations and tests. A difference between the means of the distributions of tumour-marker level in carcinoma patients and others, even if it is statistically significant, may not be useful if there is considerable overlap of the distributions. Fig. 1 illustrates this.

This was found to be the case when the distribution of CEA in urine was examined in patients with and without carcinoma. The important factor is always the use to which any difference may be put, rather than merely the establishment that there is a difference.

FIG. 1.—Distributions of tumour-marker levels in a sample free from tumour (-----, mean 1) and with tumour (——, mean 2). Black area: overlap of distributions.

*Clinical situation*

It is necessary to examine in detail the clinical situation for which the use of the tumour marker is proposed; as, for example, a diagnostic aid, or in patient monitoring.

*Diagnosis*

The diagnostic problem must be carefully assessed. It should be shown that a diagnostic problem actually exists, and that this is potentially solvable by a tumour-marker test. This is not at all a straightforward situation, as by definition these patients must be "patients suspected of suffering from cancer". The reasons for the suspicions will vary. Immediately one is in the difficult area of differential diagnosis, involving the quantification of the likelihood that a certain patient is, in the clinician's mind, suffering from one of several possible diseases.

However, at the stage when the setting-up of a study into the use of a tumour marker in differential diagnosis is under consideration, there must be some consensus of opinion on the definition of patients, and it must be shown that there is some diffiulty in confirming the actual diagnosis. The MRC set up 2 studies into the use of CEA as a diagnostic aid: one was for patients presenting with haematuria, and the other was for patients presenting with symptoms of pancreatic disease. The haematuria patients were easy to define, but in fact there did not appear to be a diagnostic problem: 227 patients were entered into the study and for 225 a firm diagnosis was made at the first hospital visit without the aid of a CEA test. The patients entered into the pancreatic study were more difficult to define, and there did appear to be a diagnostic problem (*i.e.* distinguishing between patients with carcinoma of the pancreas and benign disease). For a report of this study see M.R.C. (1980).

A review of exactly how the diagnostic problem is solved currently is of value at this stage. Establishing the diagnosis may span some period of time; the mechanism by which the diagnosis is made, the tests carried out, their reliability, cost, contribution to making the diagnosis, and time to complete are all important, as any new test is to be evaluated against this background. This may be information which is not readily available and will require some preliminary research. A tumour-marker test could fulfill a useful function by replacing an expensive or rare piece of equipment or a diffiuclt procedure, at a much cheaper cost, provided it gives results which can be interpreted in the same way.

The incidence of the cancer in question, the proportion of those patients with diagnostic difficulties and the number of patients with non-malignant disease for whom there exists a diagnostic problem should be assessed. These numbers will be needed if an objective estimate of the benefit from the use of a new test in the general population is to be made. The problem may be represented by a simple Venn diagram (Fig. 2).

The set outlined with a bold line is the one which would benefit from an additional diagnostic test, and it is relevant to examine the overall size of this, and the sizes of its two components. In the MRC study of the use of CEA as a help in differential diagnosis in patients with pancreatic disease, 30 patients had a final diagnosis of definite carcinoma. Of these 21 were confirmed without difficulty, and the other 9 were intially suspected of having carcinoma, which was subsequently confirmed. Fifty-three patients had a final diagnosis of pancreatitis or gallstones and 36 were confirmed immediately, without difficulty. Seven of the remaining 17 were initially suspected of pancreatic carcinoma and 10 of pancreatitis or gallstones. This information gives an estimate of the relative sizes of the sets in the diagram for this particular problem, and also suggests a possible role for the CEA test: to confirm or otherwise, the suspicion of carcinoma of the pancreas. It is relevant that no clinician overlooked the possibility of carcinoma; there was no case which was not suspected initially.

The consequences of the diagnosis should be considered. If cancer is diagnosed the following questions should be asked:

(1) What is the therapy?
(2) Is the treatment successful?
(3) What is the morbidity?
(4) What is the prognosis?
(5) Is treatment of this disease an area of current research?

A particularly pertinent question involves the consequences of earlier diagnosis; what is the likely impact in terms of therapy and prognosis? An answer to this may be speculative, but should be accompanied by realistic assessment of the likelihood of attaining a successful treatment. One use of a tumour marker may be to give particular information, such as the presence of metastases (*i.e.* help with

FIG. 2.—Venn diagram illustrating patients for whom a tumour-marker test may aid diagnosis.

staging). The relationship between the tumour-marker level and the size, distribution and activity of the tumour is very important. The tumour burden (*i.e.* total number of viable tumour cells) and the tumour-marker level may not themselves be related linearly, but some particular facet of the tumour may raise levels of the marker. The site of metastases, and their distribution, number and size in the target organ may be important. It would be necessary to investigate these points in great detail to obtain the maximum information from any study which may be set up.

### *Monitoring*

In considering the potential use of a tumour marker in monitoring patients, similar problems to those outlined above are encountered, but in some respects they are more easily dealt with. The most relevant use would be in patients followed up after apparently successful ablative treatment of a primary cancer. These patients therefore are more easily defined. The disease will have been staged at or around treatment and information on the prognosis will be available. The tumour may be likely to recur locally, to metastatize, or both, according to the natural history of the disease. A tumour-marker test would form an additional part of the routine follow-up examinations and, as with the diagnostic situation, must be assessed in this setting. A follow-up procedure should be agreed, including the frequency of examination, specification of the tests to be carried out on each occasion, and the overall period of the follow-up. This should fit in with normal clinical practice. A stable baseline of conventional tests must serve as a background against which the addition of a new test can be assessed. The costs and contributions of the conventional tests should be estimated. The role of a tumour-marker test would be to give early warning of recurrence not otherwise detectable by routine investigation at that stage. The potential use of this "lead time" should be examined, again in relation to therapy and prognosis. The possibility of therapeutic intervention on the ground of tumour-marker result alone should be considered, as a possible future development, should an initial study establish the test as useful. Any intervention should take the form of a randomized controlled trial, unless there already exists a successful treatment with negligible or no side effects.

Another possible role for a tumour marker is in monitoring the effect of treatment. A direct relationship between the marker level and the tumour response in the individual patient must be shown, and this requires some other method of measuring tumour response. The tumour marker could be used to "confirm" the success of ablative surgery for the removal of a primary carcinoma, or to monitor tumour response to longer-term treatment such as chemotherapy.

### PART 2: SOME PRACTICAL GUIDELINES

This part contains practical advice on setting up and conducting a study to assess the usefulness of a tumour marker. It is not intended to serve as a comprehensive guide to any such study, but to suggest some basic scientific principles to help with the setting up of a study and some practical advice on administration. It is complementary to Part 1.

### *Design*

The major practical difference between this type of designed prospective study and the preliminary research on the tumour marker is that the work of the clinicians and of the laboratory researchers must now be separated; *i.e.* the laboratory workers should not know the clinical details of the individual patients, and the clinicians should not know the result of the tumour-marker test. The "blindness" is absolutely essential in a study of this type to ensure that all samples and all patients are treated in the same way. The study must be objective, and the situation in which the laboratory is requested to repeat the tumour-marker test because carcinoma has been detected or, on the other hand, the clinician carries out repeat or extra tests on an individual patient because he knows the tumour-marker level is very high, must be avoided. The information on the clinical details and the laboratory results should be collected at one central office. It is only by withholding the tumour-marker result from the clinician that an objective estimate of its value as an additional test can be made.

Unlike a clinical trial, there is no straightforward statistical criterion that can be applied to calculate the number of patients required in a study of this nature. I would suggest that a minimum of 50 patients be included in a diagnostic study, to be sufficiently out of the range of "anecdotal evidence", and 200 in a monitoring study (following ablative therapy). This should allow for dividing patients if necessary, into 2 or 3 different prognostic groups and for some loss during the follow-up period. If it is possible to obtain this number in a reasonable time at one centre, it would be worth confining the study to that one centre. This would involve fewer clinicians and simplify the agreement on a baseline of tests against which the new test would be assessed (*i.e.* the normal clinical practice for that centre). The administration of the study would be easier. However, it has the disadvantage that the tumour marker is being assessed in a single clinical setting. If, as is more likely, it is not possible to obtain the required number of patients from one centre in a reasonably short time, I would suggest that the number of centres included be increased to 3 or 4 (at a maximum). Any more than 4 will render the study difficult to administer, and could lead to inconsistencies on the clinical side.

If one major laboratory is performing the assay, it is strongly advisable that all the samples are assayed by that laboratory, even though other laboratories, more geographically convenient for some centres, may also carry out the test. By channelling all the samples to one laboratory a major potential source of variability in tumour-marker results can be eliminated.

For studies assessing a tumour marker for use in diagnosis the patients must be carefully defined. This has been discussed in detail previously. Consecutive eligible patients should all be entered into the study.

For studies as a monitoring aid, patient definition is more straightforward. Again, all eligible patients should be included in the study. The follow-up policy, including timing of visits, tests to be carried out at each visit and total length of follow-up, must be specified in detail. If one centre sees patients annually, and another sees them six-monthly, the latter will detect recurrences earlier. The data to be collected concerning other diseases and treatments during the surveillance period must be considered. This is particularly relevant for middle-aged and elderly patients, who are likely to suffer from other diseases when followed over a considerable time period. A patient who has been admitted to hospital several times for another reason is likely to have some examination related to carcinoma as an "extra", because the history of carcinoma is known. This type of patient will be scrutinized more closely than one who only attends for prescribed routine follow-up.

*General administration*

Forms should be well designed and contain the complete information for a particular patient at a given examination (which may of course include a non-routine test, and space should be available on the form for this). There should be separate forms for the laboratory and clinical data, with unambiguous labelling to avoid any unidentified data. As stated previously, all forms should be sent to one central office. This may be situated at one of the centres involved, though it should be completely separate from the clinicians dealing with patients there. Additional staff may be required to deal with the extra workload imposed by a centre's participation in a study. The person responsible for completing the forms at each centre should be familiar with the principles behind the study and understand the information collected. If there are staff changes, any new person should be well informed about the study.

The transport of samples from the centres to the laboratory must be well organized, with a clear labelling system.

Any major investigation to be set up should be preceded by a small-scale pilot study, to ensure the smooth working of the system, to check that the study's requirements fit in with the normal clinical practice, and note any deviations, and to estimate patient accrual. Any serious differences between a hospital's normal practice and the study's requirements may require the redesign of part of the study. A pilot study offers an opportunity to establish communication channels between the laboratory, contributing hospitals and the central office. If possible, the pilot study should run straight into the major investigation, thereby retaining as much as possible of the initial data.

*Analysis*

The data-handling, coding, storage and analysis depend very much on the details of the individual study, and therefore can be dealt with only briefly here. Unless the intention is to collect and analyse a great deal of data on each patient, it is usually not worth using a computer for less than 100 patients; a system involving coding on to cards should suffice. For more than 100 patients it may be worth setting up a system for computer storage, with the facility to add extra follow-up information on each patient as it becomes available.

For the analysis, some type of cost/benefit analysis should be carried out. The total cost of the test should be estimated and the benefit which may be achieved investigated. It is not enough to show an association between level of tumour marker and the presence of disease. The benefit should assess what use the tumour-marker result could be put to, to augment what is known of the state of the patient at a given time, in the knowledge of the fate of the patient. For example, although an upper limit for the normal value of the tumour marker may be accepted, it may be valuable to experiment with other values, to obtain a cut-off point which gives more useful results. If a false positive is much less desirable than a false negative, one may choose a high critical level. This may be especially important if therapeutic action is to be taken, using chemotherapy with undesirable side-effects. If it is found that a "normal" patient occasionally has a spurious high value of the tumour marker, it may be useful to consider two consecutive high readings, or two high readings within a certain time as an indicator of malignancy. By devising simple, unambiguous decision rules, and looking at the outcomes they predict compared with the actual outcomes, different ways of using the tumour-marker result can be assessed. In general, the analysis involves examining the best use to which a tumour-marker test result may be put, and this may be different for different clinical situations. A more detailed account of alternative approaches to the analysis of this type of data, illustrated by practical examples, is to form the subject of a future publication.

### DISCUSSION

In this paper the problems involved in assessing tumour markers have been described in detail. The main conclusions are that this sort of research must be carried out well, and that this is difficult. The feasibility of any study needs careful consideration and if it is decided to go ahead, a good deal of preliminary research will be required. The practical difficulties of carrying out such a study are described. The analysis depends on the details of the individual study but in general involves examining the reward from the best use of the tumour-marker results.

A recent consensus statement (*Br. Med.*

*J.*, 1981) on the use of CEA for monitoring patients after surgical removal of colorectal carcinoma states "The regular and sequential assay of plasma CEA is the best presently available non-invasive technique for post-operative surveillance of patients to detect disseminated recurrence of colorectal cancer." This implies that a CEA test alone is all that is needed for the surveillance of these patients, and that there are no false-negative results. It goes on to say "In a substantial number of patients CEA values also become significantly raised before metastatic disease can be detected by clinical or other diagnostic measures." What exactly is meant by the word "substantial" is not stated and an account of exactly what other diagnostic measures were taken is not given. Later, a statement admits the existence of false negatives, but again no indication of the proportion of these is given: "Furthermore, some patients with recurrence or advanced colorectal cancer may not show raised plasma CEA value." These vague and almost contradictory statements are counterproductive to the general acceptance of a new test. It is not proposed that a new diagnostic test should be used alone, but in junction with other tests, and this involves the scientific examination of a complex medical situation. Any study set up to assess a tumour marker must be well designed and executed if it is to give a meaningful and unambiguous answer.

### REFERENCES

Br. Med. J. (1981) Carcinoembryonic antigen: Its role as a marker in the management of cancer. Summary of an NIH consensus statement. *Br. Med. J.*, **282**, 373.

Cole, P. & Morrison, A. S. (1978) Basic issues in cancer screening. *UICC Workshop on Screening, Toronto.* UICC Tech. Rep. Series, Vol. **40**.

Gold, P. & Freedman, S. O. (1965) Demonstration of tumor-specific antigens in human colonic carcinomata by immunological tolerance and absorption techniques. *J. Exp. Med.*, **121**, 439.

Martin, F. & Martin, M. S. (1970) Demonstration of antigens related to colonic cancer in the human digestive system. *Int. J. Cancer*, **6**, 352.

M.R.C. Tumour Products Committee (1980) The diagnostic value of plasma carcinoembryonic antigen (CEA) in pancreatic disease. *Br. J. Cancer*, **41**, 976.

WHO (1978) Guidelines for the preparation and establishment of reference materials and reference reagents for biological substances. *WHO Tech. Rep. Series No.* 626, p. 101.